# Construction of a Shuttle Vector Using an Endogenous Plasmid From the Cyanobacterium Synechocystis sp. PCC6803

**DOI:** 10.3389/fmicb.2018.01662

**Published:** 2018-07-24

**Authors:** Haojie Jin, Yan Wang, Adam Idoine, Devaki Bhaya

**Affiliations:** ^1^Department of Plant Biology, Carnegie Institution for Science, Stanford, CA, United States; ^2^Department of Neurosurgery and Stanford Stroke Center, Stanford University, Stanford, CA, United States

**Keywords:** endogenous plasmid, expression vector, synthetic biology, copy number estimation, YFP

## Abstract

To advance synthetic biology in the photosynthetic cyanobacterium *Synechocystis* sp. PCC6803 (Syn6803), we constructed a shuttle vector with some versatile features. This shuttle vector, pSCB-YFP, consists of a putative replicon identified on the plasmid pCC5.2, the origin of replication of pMB1 from *E. coli*, as well as the YFP reporter gene and a spectinomycin/streptomycin resistance cassette. pSCB-YFP is stably maintained in Syn6803M (a motile strain that lacks the endogenous pCC5.2) and expresses YFP. In addition, we engineered a fragment into pSCB-YFP that has multiple cloning sites and other features such that this plasmid can also be used as an expression vector (pSCBe). The shuttle vector pSCB-YFP can be stably maintained for at least 50 generations without antibiotic selection. It is a high copy number plasmid and can stably co-exist with the RSF1010-based pPMQAK1-GFP.

## Introduction

Cyanobacteria are an ancient and diverse phylum that play an important role in the Earth’s carbon and nitrogen cycles. The first cyanobacterial genome sequence from *Synechocystis* sp. PCC6803 (Syn6803) was released in 1996 (Kaneko et al., 1996). Based on its ability to obtain energy through photosynthesis, researchers are interested in developing cyanobacteria, Syn6803 in particular, to produce biofuels or high-value products (Huang et al., 2010; Heidorn et al., 2011; Lindblad et al., 2012; Xue et al., 2014). It is also being used as an intermediate to establish technologies to enable C4 plant bioengineering (Price et al., 2008), or to develop a nitrogen fixing photosynthetic organelle (Mueller et al., 2016). However, Syn6803 is still far from its promise as green *E. coli* (Branco dos Santos et al., 2014). Molecular genetics of Syn6803 is hampered by a lack of molecular tools, particularly the limited number of shuttle vectors. Most plasmid replicons used in *E. coli* cannot directly be used in cyanobacteria (Taton et al., 2014).

Strain-specific shuttle vectors have been constructed using endogenous plasmid replicons combined with *E. coli* plasmid replicons (van den Hondel et al., 1980; Buzby et al., 1983, 1985; Golden and Sherman, 1983; Deng and Coleman, 1999). These shuttle vectors were built before the host genomes or plasmids were sequenced, so many of them incorporated the entire endogenous plasmid which made them unwieldy to manipulate. In addition, the plasmid replicon system seems to be species-specific in cyanobacteria (Taton et al., 2014). Recently, a shuttle vector based on the endogenous plasmid pANS from *Synechococcus elongatus* PCC7942 (Syn7942) was shown to replicate in *Anabaena* PCC7120 but not in Syn6803 (Chen et al., 2016b). Identification of the plasmid replicon or plasmid located replication initiation protein (Rep protein) is challenging. Recently, a web platform dedicated to cyanobacterial synthetic biology shuttle vector assembly was developed based on four plasmid replicons (Taton et al., 2014), pDU1 from *Nostoc* sp. PCC7524 (Wolk et al., 1984); pFDA from *Fremyella diplosiphon* (Cobley et al., 1993); pDC1 from *Nostoc* sp. MAC PCC8009 (Lambert and Carr, 1983) and pANS from Syn7942 (Chen et al., 2016b). All these replicons contain an open reading frame (ORF) with homology to Rep proteins. However, these ORFs have very different lengths and low homology to each other, such that identification of further plasmid replicons based on homology is not feasible.

So far, only one non-native replicon, RSF1010, has served in Syn6803 as the basis of shuttle vectors (Huang et al., 2010). RSF1010 is a broad-host-range plasmid replicon belonging to the IncQ family, which is able to replicate in a wide range of Gram-negative strains (Meyer, 2009). Although the widely used RSF1010-based pPMQAK1 is stable, it is a low copy number plasmid present only in about 10–20 copies per cell in *E. coli*, which is a limitation to cloning and when high gene expression is required. Though Syn6803 is naturally competent and exhibits high frequencies of homologous recombination, development of multi-purpose compatible shuttle vectors would help advance the bioengineering process because Syn6803 is highly polyploid (Griese et al., 2011), which makes segregation after chromosomal homologous recombination laborious and time-consuming. Another issue for gene integration into the host genome is inherent genome instability (Jones, 2014).

There was one report in 1986 describing the construction of a shuttle vector in Syn6803, based on fragments from the endogenous plasmid pCA2.4; however, the stability of this shuttle vector made it impractical for use (Chauvat et al., 1986). Except for this report, there have been no other published attempts to create a shuttle vector for Syn6803. Syn6803 has seven native plasmids (Kaneko et al., 1996), among which, four are large (pSYSM: 120 kb, pSYSX: 106 kb, pSYSA: 103 kb, and pSYSG: 44 kb) (Kaneko et al., 2003), and three are small (pCA2.4: 2.4 kb, pCB2.4: 2.3 kb, and pCC5.2: 5.2 kb). We focused our attention on the three small plasmids pCA2.4, pCB2.4, and pCC5.2. These small plasmids were predicted to replicate through the Rolling Circle Replication (RCR) model based on the identification of single-stranded plasmid DNA in the cell (Yang and McFadden, 1993, 1994; Xu and McFadden, 1997). The same RCR model has been predicted in other cyanobacterial species based on nick site sequence similarity (Kawaguchi et al., 1994) or on the presence of single-stranded plasmid DNA in the cell (Kurokawa et al., 1994; Tominaga et al., 1995). Thus, cyanobacteria may use a different plasmid replication mechanism compared to other Gram-negative bacteria like *E. coli*, which normally use a theta structure replication mechanism (del Solar et al., 1998).

In this study, we describe the identification of a putative replicon (ORFb and its flanking region) from the endogenous small plasmid pCC5.2 in Syn6803, and the construction of a new high copy number shuttle vector (pSCB-YFP). pSCB-YFP was conjugatively transformed into Syn6803M (a motile strain that lacks pCC5.2) to express YFP. pSCB-YFP can stably exist in Syn6803M for at least 50 generations in the absence of antibiotic selection. In addition, we engineered a fragment into pSCB-YFP that has multiple cloning sites (MCS) and other features such that this plasmid can be used as an expression vector (pSCBe). We also demonstrated that pSCB-YFP and pPMQAK1-GFP are compatible and can co-exist in Syn6803M.

## Materials and Methods

### Strains and Growth Conditions

*Escherichia coli* strain DH5α was used for plasmid propagation and cloning unless otherwise stated (growth in LB medium at 37°C, 200 rpm). Syn6803GT used in this study is a non-motile spontaneous glucose-tolerant mutant Syn6803. It can grow in the dark in BG11 medium supplemented with up to 20 mM glucose (Williams, 1988), which allows this strain to grow both photo-autotrophically and heterotrophically. Syn6803M is a model strain for motility studies (Bhaya et al., 1999). Syn6803 strains were grown under 25 μE m^-2^ s^-1^ continuous illumination at 30°C in BG11 liquid medium with shaking (50 or 250 ml flask, 170 rpm) or on agar plates. For photomixotrophic growth, 5 mM glucose was added to the medium. When the strain contained a plasmid, suitable appropriate antibiotics were added at the following concentrations: 10 or 100 μg/ml Spectinomycin (Sp), 50 or 100 μg/ml Carbenicillin (Cb) and 25 or 50 μg/ml Kanamycin (Km) for Syn6803 and *E. coli*, respectively. Strains and plasmids used in this study are summarized in **Table 1**.

**Table 1:** Strains and plasmids used in this study.

Name	Purpose or relevant characteristics	Reference
**Strains**	
Syn6803GT	Spontaneous glucose tolerant mutant derivative of Syn6803	Williams, 1988
Syn6803M	Motile type strain derivative of Syn6803	Bhaya et al., 1999
DH5α	F– Φ80*lac*ZΔM15 Δ(*lac*ZYA-*arg*F) U169 *rec*A1 *end*A1 *hsd*R17 (rK–, mK+) *pho*A *sup*E44 λ– *thi*-1 *gyr*A96 *rel*A1	Thermo Fisher
SCB	Syn6803M bearing pSCB	This study
SCB-YFP	Syn6803M bearing pSCB-YFP	This study
PMQAK1-GFP	Syn6803M bearing pPMQAK1-GFP	This study
SCB-PMQAK1	Syn6803M bearing pSCB-YFP and pPMQAK1-GFP	This study
**Plasmids**	
pBR322	Ap^r^, Tc^r^, *bom+* (GenBank: J01749.1)	Balbás et al., 1986
pPZP200	Sp^r^, Sm^r^ (GenBank: U10460.1)	Hajdukiewicz et al., 1994
pGJ28	Km^r^, Conjugation helper plasmid	Van Haute et al., 1983
pRL443	Ap^r^, Tc^r^, Conjugative plasmid	Elhai et al., 1997
pSCB	Sp^r^, shuttle vector	This study
pSCB-YFP	Sp^r^, shuttle vector with YFP reporter gene	This study
pSCBe	Sp^r^, expression vector	This study
pPMQAK1-GFP	Ap^r^, Km^r^, RSF1010 replicon-based plasmid expressing GFPmut3	Huang et al., 2010


For microscopy, cultures of Syn6803 carrying the various constructs were grown in 10 ml BG11 medium supplemented with antibiotics for 3 days, then inoculated into 50 ml of medium in a 250 ml flask starting with an initial optical density of OD_730_ = 0.1 (supplemented with antibiotics, 30°C, 170 rpm), then cultured for an additional 3 days until the OD_730_ reached ∼2–2.5. For plasmid copy number assays, cells were taken at stationary phase, which took ∼5 days starting with an initial OD_730_ = 0.1.

### Total DNA Extraction From Syn6803

Fifty milliliters culture of Syn6803 were grown for 5 days, at which time the OD_730_ was ∼2.5, then collected by centrifugation at 10,000 × g for 10 min, for total DNA isolation. Cells were resuspended in 500 μl TE buffer (50 mM Tris-HCl, 10 mM EDTA, pH 8.0) in 2 ml screw cap tube (Thermo Fisher, Cat No. 3463). 0.5 g of 0.1 mm diameter glass beads (SIGMA, Cat No. G1145-500G), 25 μl of 10% sodium dodecyl sulfate (SDS), and 500 μl of phenol–chloroform–isoamyl alcohol (25:24:1, v/v) were added and cells were disrupted using a Biospec Mini-Beadbeater (Model 3110BX) with four cycles of maximum speed for 30 s, with 2 min on ice between the cycles. The liquid phase was separated by centrifugation at 14,000 × *g* for 15 min, and the upper aqueous phase was extracted with an equal volume of phenol–chloroform–isoamyl alcohol (25:24:1). The upper aqueous phase was subsequently extracted two more times with an equal volume of chloroform:isoamyl alcohol (24:1). The DNA was precipitated with 1/10 volume of 3 M sodium acetate (pH 5.2) and two volumes of 100% ethanol at -20°C for several hours (or overnight). DNA was collected by centrifugation at 4°C, 12,000 × g for 10 min, the supernatant was discarded, and the pellet washed twice with 70% ethanol. After drying, DNA was dissolved in water (Heidorn et al., 2011). DNA concentration was quantified spectroscopically with the NanoDrop 1000.

### PCR and Southern Blot

Two hundred nanograms of total DNA was used as a PCR template to test for specific plasmid presence using the primers listed in **Table 2**. Two pairs of primers, DB148 and DB149, and DB150 and DB151, were designed to amplify specific amplicons 392 bp and 778 bp long, respectively, from the plasmid pCA2.4. Two pairs of primers, DB152 and DB153, and DB154 and DB155, were designed to amplify specific amplicons 701 bp and 645 bp long, respectively, from plasmid pCB2.4. Three pairs of primers were designed based on the sequence of plasmid pCC5.2. DB156 and DB157 amplify the 651 bp region between position 147 and 797 bp for use as DNA hybridization probe 1; DB158 and DB159 amplify the 787 bp region between 1273 and 2059 bp; and DB160 and DB161 amplify the 790 bp region between 2272 and 3061 bp for use as DNA hybridization probe 2. A 660 bp fragment from 16S rRNA was amplified with primer pair DB554 and DB555 and used as a positive control for both PCR and DNA hybridization (probe 3). All these probes were generated from PCR amplification using Syn6803GT total DNA as the template. Three pairs of primers which cover different regions of pSCB-YFP (DB239 and DB159, DB160 and DB161, and DB187 and DB188) were designed to screen Syn6803 exconjugants by PCR for the presence of pSCB-YFP. Another set of PCR primers DB245 and DB246 (amplicon length: 856 bp) which cover the region of pSCB-YFP *E. coli* replication origin pMB1, was used as DNA hybridization probe 4. PCR was conducted using Phusion Hot Start II DNA polymerase (Thermo Fisher, Cat No. F549L) with initial denaturation at 98°C for 2 min, then 30 cycles with 98°C denature 15 s, 58°C annealing 30 s, 72°C extension from 15 to 30 s according to PCR product size, with a 72°C final extension step of 5 min.

**Table 2:** Oligonucleotides used in this study.

Primer name	Sequence	ID
pCA2.4-F1	GAGCAACACAAGGAACCGACAG	DB148
pCA2.4-R1	CATCCGCGACTTATCCCTCAGT	DB149
pCA2.4-F2	TTACGACTCCTTAGCGGGCAG	DB150
pCA2.4-R2	GTTATCAGGCTCGGAGTCGTCA	DB151
pCB2.4-F1	TGGGCAGTGTCGCAAGTAGTTC	DB152
pCB2.4-R1	CCAGAAGTCGAGACACCACCAT	DB153
pCB2.4-F2	GGTCACAGTTTCGGCAAGTTCC	DB154
pCB2.4-R2	CACTTTCCATCAGGCCCAACC	DB155
pCC5.2-F1	GTAAACGCTTATCCTGCCCTGC	DB156
pCC5.2-R1	CAGAGCCCGTCAGCAAAGTCTT	DB157
pCC5.2-F2	TTGACAAAGGGATAGCGACAGC	DB158
pCC5.2-R2	CATCTTCCGGTGGTTTGACCC	DB159
pCC5.2-F3	AAGGGTAGCAAGGGACGAAAGG	DB160
pCC5.2-R3	CCTGCACTGGCTTAAACGTG	DB161
GB^a^-ORFb-F	GAGGCCCTTTCGTCTTCAAGAAACAGCGTGACAGCGACCGATC	DB202
GB-ORFb-R	CCTGCATGGGTTTAGCCTGTTA	DB203
GB-pBR322-F	TAACAGGCTAAACCCATGCAGGCAAACCAACCCTTGGCAGAAC	DB241
GB-pBR322-R	GCCAAACTATCAGGTCAAGTCTGTCAGACCAAGTTTACTCA	DB242
GB-Sp-F	ACTTGACCTGATAGTTTGGCTGTG	DB213
GB-Sp-R	GTCACGCAACTGGTCCAGAA	DB214
pSCB-YFP-seq1	AAACAGCGTGACAGCGACCGATC	DB177
pSCB-YFP-seq2	CAAGCCAAGGAAGACAGGCAG	DB181
pSCB-YFP-seq3	Same as pCC5.2-F3	DB160
pSCB-YFP-seq4	CCCGGTGACTATCAAGTATGTCC	DB183
pSCB-YFP-seq5	GGGAAGAAGGGTACACACAGAAAG	DB184
pSCB-YFP-seq6	ATCGCCTTCGTGCAATCTGTC	DB187
pSCB-YFP-seq7	AACCCGTATCGTGAGCATCC	DB244
pSCB-YFP-seq8	TCAAGTCAGAGGTGGCGAAAC	DB245
pSCB-YFP-seq9	GAAAGGCGAGATCACCAAGG	DB246
pSCB-YFP-seq10	GTAACGCGCTTGCTGCTTGG	DB243
pSCB-YFP-seq11	AGCAGAAGAACGGCATCAAGG	DB239
pSCB-YFP-R	GAAACACGGAAACCGAAGACCA	DB188
Q^b^-petB-F	CCTTCGCCTCTGTCCAATAC	DB219^c^
Q-petB-R	TAGCATTACACCCACAACCC	DB220^c^
Q-16sRNA-F	CACACTGGGACTGAGACAC	DB231^c^
Q-16sRNA-R	CTGCTGGCACGGAGTTAG	DB232^c^
Q-pSCB-YFP-F	TCAAGCCAAGGAAGACAGG	DB423
Q-pSCB-YFP-R	CTGTCGGTGCTAGATACTGC	DB424
Q-bla-F	CTACGATACGGGAGGGCTTA	DB419^d^
Q-bla-R	ATAAATCTGGAGCCGGTGAG	DB420^d^


*^a^Primers with GB- initials mean they have overlap sequences for Gibson assembly. ^b^The primers with Q- initials mean they are qPCR primers for plasmid copy number assay. ^c^Cited from Pinto et al. (2012). ^d^Cited from Lee et al. (2006).*

All DNA hybridization probes were labeled using GE Healthcare Amersham Gene Images AlkPhos Direct Labelling and Detection System kit, among which probe 1 and probe 2 were used to establish the presence of plasmid pCC5.2, and probe 2 and probe 4 were used to screen for pSCB-YFP plasmid in Syn6803M exconjugants. Probe 3 which hybridized to the 16S rRNA gene was used as a control in both tests. All probes used in this study were highly specific for target fragments; NCBI blast using these probes did not generate any non-specific alignments. For DNA hybridizations, 2 μg of overnight enzyme-digested total DNA was loaded onto 1% agarose gels and run at 6 V/cm for 2 h. Different enzymes were used for the DNA digestions, EcoRV was used for plasmid pCC5.2, PstI was used for plasmid pSCB-YFP, and HindIII used for Syn6803 genomic DNA (Chen et al., 2016a). Southern blot hybridization was performed using GE Healthcare Amersham Gene Images AlkPhos Direct Labelling and Detection System and Amersham CDP-Star Detection Reagent kits.

### Construct Preparation

The pSCB-YFP shuttle vector was assembled from four fragments. These fragments were obtained by PCR or synthesized by Integrated DNA Technologies (IDT) with 20 bp overlapping sequences of neighboring fragments for Gibson assembly (Gibson et al., 2009). The pCC5.2 plasmid ORFb which is 2916 bp including 5′ and 3′ untranslated regions (257 and 69 bp, respectively) was amplified using primers DB202 and DB203. A fragment containing the origin of replication (pMB1) and *bom* site (oriT) from pBR322 was amplified using primers DB241 and DB242. The *bom* site was retained to ensure the shuttle vector can be conjugatively transformed with the helper plasmid pGJ28. The *aadA* gene which confers Sp resistance was amplified with primers DB213 and DB214 using plasmid pPZP200 as a template. *pSCB-YFP Synthesis fragment* which contains the MCS, the Trc1O promoter (Huang et al., 2010), the synthetic ribosome binding site (RBS) (BBa_B0034), YFP (BBa_E0030), and the terminator (BBa_B0015) was synthesized by IDT (Supplementary Figure 1). The components of RBS, YFP, and terminator which begin with “*BBa_*” were taken from iGEM Registry of Standard Biological Parts^1^. The four fragments were assembled by Gibson assembly (NEB, Cat. No. E2611L). The whole assembled shuttle vector was verified by sequencing using primers (pSCB-YFP-seq1 to 11) shown in **Table 2**. The pSCB-YFP construct was digested by XbaI and SpeI which generated compatible sticky ends, and ligated with T4 ligase. This generated the pSCB construct which was verified by sequencing using primer DB243. pSCB which does not contain the YFP reporter gene was used as a negative control for confocal microscopy to examine YFP fluorescence. For the generation of the expression vector pSCBe, pSCB-YFP was digested with NotI and SpeI as the plasmid backbone, then the *pSCBe-MCS* fragment which was synthesized by IDT with different components (detailed annotation in **Figure 3A**) was assembled with plasmid backbone through Gibson assembly, and then verified by sequencing with primer DB243.

### Conjugation

The conjugation procedure was based on the method described by Elhai and Wolk (1988). pGJ28 and pRL443 were used as helper and conjugative plasmids, respectively. All the Syn6803 exconjugants were grown on antibiotic-free LB agar plates to confirm the absence of *E. coli* contamination, and verified by sequencing before they were used for further experimental procedures in the study.

### Leica TCS SP8 Confocal Microscopy

Cells were observed with the Leica TCS SP8 60x oil objective. The excitation and emission wavelength for different fluorescent reporters were set as chlorophyll autofluorescence (Ex: 550 nm; Em: 650–700 nm) and YFP (Ex: 500 nm; Em: 540–575 nm).

### Fluorescence Characterization by a Plate Reader

YFP fluorescence was assayed using the TECAN Infinite M1000 PRO Microplate Reader, with 360 rpm shaking (2 mm amplitude) for 10 s. The YFP reporter was excited at 515 ± 5 nm and emission measured at 530 ± 5 nm; while OD_730_ was also measured for normalization. For each sample, 120 μl of culture was transferred into a Greiner 96 Flat Bottom Transparent Polystyrene Microplate (Sigma-Aldrich Cat. No: 655101). Each sample was measured using three biological and three technical replicates. The YFP signals of all the exconjugants were normalized by dividing the signal by OD_730_ and subtracting the signal of the negative control.

### Plasmid Stability Assays and Data Analysis

Maintenance of the plasmid in the absence of antibiotic selection was assayed based on the shuttle vector maintenance protocol in Syn7942 with some minor modifications (Chen et al., 2016b). The test was conducted at a similar stage (1-week-old cultures) because Syn6803 and Syn7942 have similar doubling times (between 7 and 12 h) (Bernstein et al., 2016). Cells with pSCB-YFP were grown in medium supplemented with Sp 10 μg/ml for 7 days, then inoculated into two flasks, each containing 50 ml of medium, one containing 10 μg/ml Sp, the other without antibiotic. Each treatment was performed for three biological replicates. Syn6803M that had no plasmid was used as the negative control. After 7 days of growth (OD_730_: 2.5–3), these cultures were tested for YFP fluorescence using a TECAN Infinite M1000 PRO Microplate reader. Each culture was used as a seed culture for the next iteration of the same procedure for 5 weeks.

### Quantitative PCR to Assay Plasmid Copy Number

To measure the ratio of shuttle vector pSCB-YFP to chromosome copy number, we developed the following protocol. In order to minimize variability in the purification of genomic and plasmid DNA molecules that have different structures and sizes, we directly used clones as templates to assess the plasmid copy number, as follows. A 2 ml sample of each stationary phase culture was centrifuged at 13,000 × *g* for 10 min, and the pellet was suspended in 120 μl RNase/DNase-free water, and heated at 97°C for 10 min, then put on ice for immediate usage or at -20°C for long-term storage. Real-time quantitative PCR (qPCR) was used to assay the ratio of plasmid to chromosome copy number in these crude extracts. The ratio was calculated based on the equation:

$$\begin{array}{lll} \text{Copy number ratio} & = & \frac{\text{Plasmid}}{\text{Chromosome}}\\ & = & \frac{\text{Average signal from plasmid primers}}{\text{Average signal from chromosomal primers}} \end{array}$$

Two target regions were selected, one is on pSCB-YFP ORFb (DB423 and DB424) which generated a 122 bp amplicon; the other is on the β-lactamase gene (*bla*) (DB419 and DB420) which is located on pPMQAK1-GFP and generated an 81 bp amplicon (Lee et al., 2006); two reference genes were selected from the chromosome (Pinto et al., 2012), *petB* (DB219 and DB220) which is single copy and generated a 179 bp amplicon, and the 16S rRNA gene (DB231 and DB232) which has two copies and generated a 190 bp amplicon. SensiFAST^TM^ SYBR No-ROX kit from BIOLINE (Cat. No. BIO-98020) was used for qPCR. All primers are listed in **Table 2**. qPCR was performed on Roche LightCycler^®^ 480 Multi-well in 96-well plates covered with Optical Sealing Foils (Roche Cat. No. 04729749001). Data analysis was conducted based on LinRegPCR software (Ruijter et al., 2009). The PCR efficiencies were calculated from the algorithm of LinRegPCR which can bypass the need for standard curve method to calculate PCR efficiencies. All primer pairs showed PCR efficiencies from 0.93 to 0.94. The average signal used for plasmid copy number ratio calculation was the extracted N_0_ value which is the target quantity or starting concentration per sample expressed in arbitrary fluorescence unit. All reported results are from two biological and three technical replicates.

## Results and Discussion

### Identification of ORFs and Possible Replicons in the Three Small Plasmids of Syn6803

To develop the new shuttle vector, we focused on the three small plasmids in Syn6803 to identify possible replicons. There are a total of eleven putative ORFs on the three small plasmids: two on pCA2.4; three on pCB2.4 and six on pCC5.2. ORF1 on pCA2.4 (336 amino acids [aa]), shows 52% identity with the Rep protein from *Tolypothrix bouteillei* (WP_038092011); 51% identity with plasmid Rep protein from *Planktothrix agardhii* (CUM62448) and *Synechococcus* sp. PCC 8807 (ANV92011); ORF2 on the pCA2.4 (101 aa) does not show any similarity to annotated sequences in the NCBI database. The pCB2.4 plasmid ORF1 (392 aa) shows 25% and 24% identity with “replication initiation factor domain-containing protein” from *Geobacillus thermoleovorans* (WP_083221596.1) and *Bacillus cereus* (WP_080467820.1), respectively; ORF2 and ORF3 on pCB2.4 (124 and 92 aa, respectively) do not show any sequence similarity to known proteins. Most of the ORFs on pCC5.2 (**Table 3**) are annotated as hypothetical proteins but a region between 163 and 272 aa on ORFb shows 39% similarity with a TOPRIM-like domain which suggests a function related to DNA primase or topoisomerase for DNA replication. No experimental evidence is available to confirm any of these putative functions.

**Table 3:** Endogenous pCC5.2 plasmid ORFs.

ORF name	ID in GenBank^a^	Co-ordinates^b^	ORF size (bp/aa)	Predicted function
ORFa	MYO_620	D (692–1045)	354/118	Hypothetical protein
ORFb	MYO_630	D (1600–4515)	2916/972	39% DUF3854 domain-containing protein
ORFc	MYO_640	D (4578–4859)	282/94	Hypothetical protein
ORFd	MYO_650	D (4861–5136)	276/92	38% acyl-CoA synthetase
ORFe	In ORFb	C (3712–4017)	306/102	No annotation
RFf	MYO_610	C (3–296)	294/98	Hypothetical protein


*^a^GenBank accession: CP003272.1. ^b^D, direct; C, complementary.*

**FIGURE 1:**
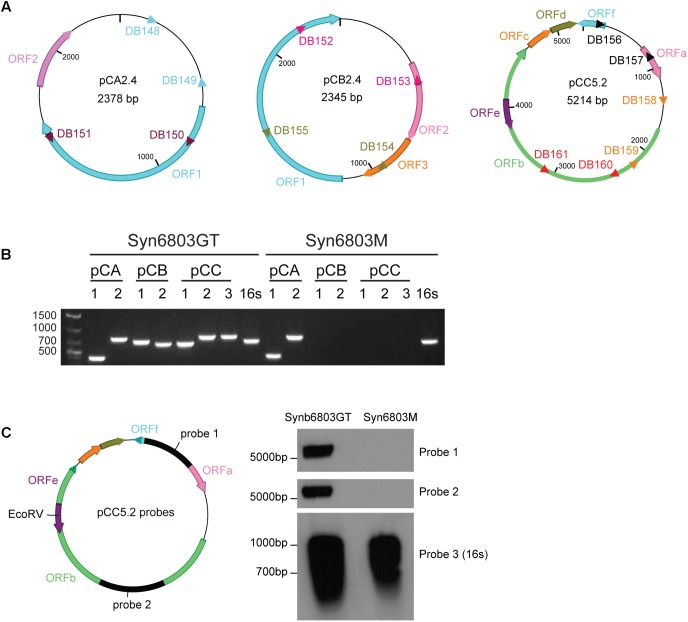
The absence of pCC5.2 plasmid in Syn6803M. **(A)** Schematic of the three native small plasmids from Syn6803. ORFs on different plasmids were shown as color arrows. pCA2.4 is a 2378 bp plasmid with two predicted ORFs, ORF1 and ORF2. pCB2.4 is a 2345 bp plasmid with three predicted ORFs (1, 2, and 3). There are six predicted ORFs on pCC5.2 with ORFe contained within ORFb. PCR primer sets for the plasmid presence assay were shown as different color triangles. Primer pairs DB148 and DB149, and DB150 and DB151 on pCA2.4, DB152 and DB153, and DB154 and DB155 on pCB2.4, DB156 and DB157, DB158 and DB159, and DB160 and DB161 on pCC5.2 were designed based on plasmid sequences. **(B)** PCR screening for the presence of three small plasmids in Syn6803GT and Syn6803M based on primer sets from **A**. Primer sets (pCA 1, 2) which generated amplicons 392 and 778 bp from pCA2.4, primer sets (pCB 1, 2) which generated amplicons 701 and 645 bp from pCB2.4, primer sets (pCC 1, 2, 3) which generated amplicons 651, 787, and 790 bp from pCC5.2. All primer sets produced positive amplicons from Syn6803GT and were verified by sequencing. In Syn6803M, only the two amplicons from pCA2.4 were revealed. No amplicons from pCB2.4 or pCC5.2 were generated. Primer pair DB554 and DB555 which amplify a 660 bp fragment from 16S rRNA gene was used as a PCR positive control (16s) for both strains. **(C)** Probes (probe 1 and probe 2) for DNA hybridization assay of the pCC5.2 plasmid were generated from primer sets DB156 and DB157 (probe 1), and DB160 and DB161 (probe 2). Total DNA was digested with EcoRV which is a unique enzyme site on pCC5.2 and can be used for the plasmid presence assay. DNA hybridization on Syn6803GT total DNA, with both probes, generated bands around 5 kb, which is the expected linear size of pCC5.2. No band was obtained from Syn6803M total DNA, as expected. 16S rRNA probe (probe 3) was used as Southern blot positive control for both strains.

Based on sequence similarity alignment, ORF1 on pCA2.4 (336 aa), ORF1 on pCB2.4 (392 aa) and ORFb on pCC5.2 (971 aa) are potential candidates as plasmid Rep proteins (**Figure 1A**), though it is not mandatory that each plasmid needs its own Rep protein for replication (del Solar et al., 1998). There are four characterized cyanobacterial plasmid replicons which have successfully been used as replicon components in building a vector (Taton et al., 2014). All these replicons contain ORFs with very different amino acid lengths; the ORF from *Nostoc* sp. PCC7524 pDU1 is 373 aa; from *Fremyella diplosiphon* pDFA is 435 aa; from *Nostoc* sp. MAC PCC8009 pDC1 is 344 aa; and from Syn7942 pNAS is 876 aa. The Rep protein candidates on the three small plasmids of Syn6803 also have very different lengths, ranging from 336 aa in pCA2.4 to 971 aa in pCC5.2. In addition, nucleotide analysis of pCA2.4 identified a predicted promoter **TTGACA**AAGTAGAAACCCTCTAGC**TAAGCT**, located 42 bp upstream and a Shine-Dalgarno (SD) sequence (AGAGGG) 7 bp upstream of the start codon of ORF1 (Yang and McFadden, 1993). In pCC5.2, ORFb, there is a Pribnow box (TATTGT) (-10 region) and an SD located 62 bp and 5 bp upstream of the start codon, respectively. However, no typical consensus -35 region was found in the 5′ UTR region of ORFb (Xu and McFadden, 1997). A similar situation was also observed for other ORFs on pCC5.2. The lack of a typical -35 region might indicate that these promoters belong to the cyanobacteria type II promoter category (Camsund and Lindblad, 2014). In addition, there are three predicted double-strand origin nicking sites on pCC5.2 (Xu and McFadden, 1997), which are all located within ORFb (CTTGATT: 2103–2109 bp; 3670–3676 bp; 4504–4510 bp) and might represent Rep protein binding sites for plasmid replication initiation. However, no experimental evidence for the role of ORFb is currently available.

### Presence of Small Plasmids in the Different Syn6803 Lineages

It was observed in the 1980s that one of the small plasmids, pCC5.2, could be spontaneously cured from both motile and non-motile strains of Syn6803 (Castets et al., 1986). In another motile Syn6803 sub-strain PCC-M, Trautmann et al. (2012) detected the pCB2.4 and pCC5.2 by PCR amplification, but not by genome sequencing and suggested that they might be present at low copy numbers. There are several strains of Syn6803 maintained in different laboratories (Ikeuchi and Tabata, 2001), so we tested two commonly used strains of Syn6803, Syn6803GT, and Syn6803M, for the presence of the three small plasmids (pCA2.4, pCB2.4, and pCC5.2). This was determined by PCR, using multiple primer sets to amplify specific plasmid fragments from isolated total DNA. The result showed that fragments specific to all three plasmids were amplified from Syn6803GT (also verified by sequencing). Only pCA2.4-specific fragments were amplified from Syn6803M (**Figure 1B**).

The initial results based on PCR amplification indicated that pCC5.2 is absent from Syn6803M. To corroborate this observation, we used a DNA hybridization based approach. DNA hybridization on total DNA from both Syn6803GT and Syn6803M strains was conducted using two probes (probe 1 and probe 2) which are located in different regions of pCC5.2 (**Figure 1C**). As shown in **Figure 1C**, pCC5.2 could be detected in Syn6803GT with both probes. In contrast, no signal was detected from total DNA extracted from Syn6803M, confirming that pCC5.2 is absent from Syn6803M. The plasmid pCC5.2 seems dispensable for cell survival, but it is unclear whether this endogenous plasmid has been spontaneously lost or if it was never present in strain Syn6803M. However, the absence of pCC5.2 from Syn6803M suggests it can be used as a host to test a shuttle vector based on the pCC5.2 plasmid, since the shuttle vector would not have to compete with the endogenous plasmid, and interference by recombination would also be avoided.

### Construction of the Shuttle Vector pSCB-YFP

**FIGURE 2:**
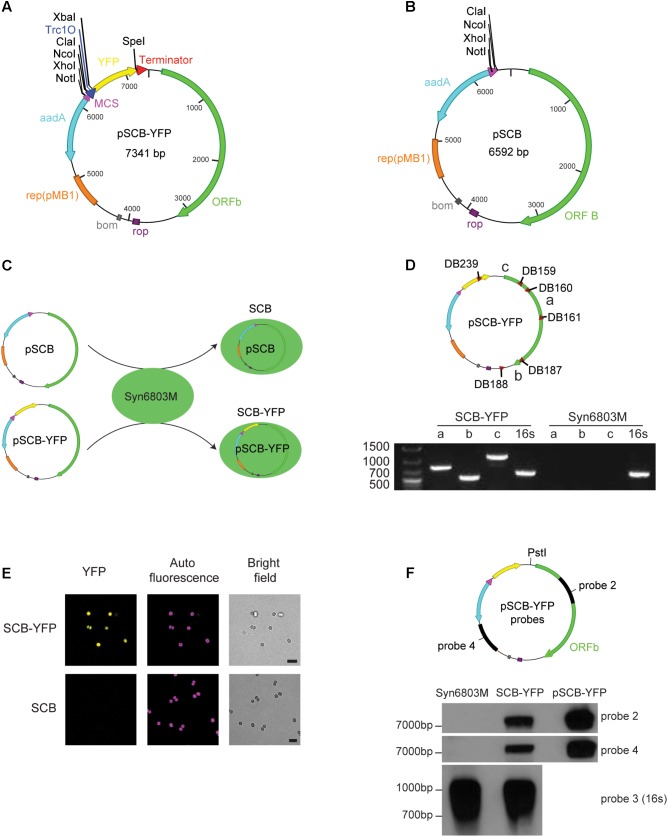
pSCB-YFP shuttle vector built and its stability assay. **(A)** pSCB-YFP shuttle vector was built to test whether ORFb and its flank region can support plasmid replication in Syn6803. This plasmid contains the *E. coli* pMB1 origin of replication (orange bar); the rop gene (purple bar), which encodes a restrictor of plasmid copy number; *bom* site for conjugative transformation (gray box); Sp cassette (*aadA*) for spectinomycin selection (light blue arrow); The YFP reporter gene (yellow arrow) was driven by a Trc1O promoter (blue triangle), and an XbaI site downstream of the Trc1O promoter and a SpeI site right before the B0015 terminator (red triangle) were also added. For cloning, a multiple cloning sites (MCS; purple triangle) was introduced which contains four unique enzyme sites as listed. **(B)** pSCB has similar components as pSCB-YFP except YFP gene was removed from the plasmid. This vector was used as negative control for SP8 confocal microscope observation. **(C)** pSCB and pSCB-YFP plasmids were conjugated into Syn6803M, generating stains SCB and SCB-YFP, respectively. **(D)** Three pairs of primers were designed for pSCB-YFP to test the presence of the plasmid in SCB-YFP exconjugants. Primer sets DB160 and DB161 (amplicon a), DB187 and DB188 (amplicon b), and DB239 and DB159 (amplicon c) can amplify bands of 790, 545, and 1109 bp, respectively. SCB-YFP showed positive bands for all three primer sets which were verified by sequencing, while no bands were detected from wild-type Syn6803M, as expected. 16S rRNA primers were used as PCR positive control for both strains. **(E)** Observation of SCB-YFP and SCB exconjugants with Leica SP8 confocal microscope under YFP, DsRed and bright field channels. We can see that SCB-YFP showed YFP fluorescence under the channel, while SCB negative showed nothing. Exconjugants’ auto-fluorescence and bright field pictures are shown alongside the fluorescence images. Scale bar indicates 5 μm in length. **(F)** Two probes (probe 2 and probe 4) were used to test pSCB-YFP stability in SCB-YFP exconjugants. Probe 2 is the same as in **Figure 1C**, probe 4 is a PCR amplicon based on primer set DB245 and DB246 using pSCB-YFP as a template. PstI which is the unique enzyme site on pSCB-YFP plasmid was used for total DNA digestion. PstI digested pSCB-YFP plasmid from *E. coli* was used as a control to indicate the size of the intact pSCB-YFP vector. 16S rRNA probe which is the same as in **Figure 1B** was used as Southern blot positive control. DNA hybridization results showed that no hybridization was detected in the Syn6803M negative control, and a band corresponding to the size of linear pSCB-YFP was detected in SCB-YFP strain with both probes.

As shown in **Table 3**, there are six putative ORFs in pCC5.2; of these, ORFb (971 aa) contains a 110 aa region with homology to the TOPRIM (DNA primase or topoisomerase)-like domain, and a potential nick site to initiate plasmid replication. Therefore, we tested if ORFb (2916 bp) together with its 5′ and 3′ UTR (257 bp and 69 bp, respectively), could work as a replicon to support plasmid replication in Syn6803. The new shuttle vector pSCB-YFP, which is 7341 bp in length (**Figure 2A**), was built as described in Materials and Methods. We used the Trc1O promoter (63 bp), which is a strong constitutive promoter (Huang et al., 2010) to drive the YFP reporter gene. pSCB (6592 bp) without the YFP reporter gene was used as a control (**Figure 2B**). Conjugative transformation of these two plasmids into Syn6803M was performed, which generated exconjugants SCB-YFP and SCB (**Figure 2C**), respectively. Single colony exconjugants (after removal of *E. coli* contamination), could grow stably on Sp (10 μg/ml) BG11 agar plates. These results suggested that the 3242 bp fragment from pCC5.2 could support plasmid propagation in Syn6803.

To establish whether pSCB-YFP was stably maintained, PCR was conducted on putative exconjugants to check if the pSCB-YFP shuttle vector is present in SCB-YFP strains. All putative colonies returned positive PCR results which were verified by sequencing (**Figure 2D**). Next, fluorescence output was observed using confocal microscopy. Cells containing pSCB-YFP showed strong YFP fluorescence, whereas cells containing the pSCB control did not (**Figure 2E**). To establish that pSCB-YFP is a stable shuttle vector which does not undergo post-transformation recombination, total DNA from SCB-YFP exconjugants was isolated and used to conduct Southern blot DNA hybridization using two different probes (**Figure 2F**). The pSCB-YFP plasmid isolated from *E. coli* (linearized with PstI, 7341 bp) was used as the positive control. Two separate probes identified fragments of the expected size (7341 bp) from the total DNA derived from exconjugants (**Figure 2F**). This indicates that the shuttle vector can stably exist in the cell for several generations, without recombining into the host DNA. This attribute is important for its successful use as a shuttle vector. These results support the conclusion that ORFb along with its flanking regions from pCC5.2 (3242 bp) can function as a replicon to support plasmid replication in Syn6803M. However, the mechanism of plasmid replication, and whether ORFb is an authentic Rep protein requires further study.

The shuttle vector pSCB-YFP was verified by sequencing and deposited in Addgene’s non-profit plasmid repository under ID 101737 for community use. The entire plasmid sequence has been submitted to Genbank (accession number MH144608). The replicons for *E. coli* and Syn6803 on pSCB-YFP support vector propagation in both species. The four unique enzyme digestion sites upstream of Trc1O promoter can be used to clone specific DNA fragments. So far, attempts to transfer pSCB-YFP into Syn6803GT have been unsuccessful. This may be because of the presence of the resident pCC5.2 which prevents stable maintenance of pSCB-YFP or for other reasons that we have not explored in this study. In Syn7942, curing of endogenous plasmids has been successful (Kuhlemeier et al., 1983; Chen et al., 2016b), it is possible that curing of resident pCC5.2 from Syn6803GT may allow for successful maintenance of pSCB-YFP.

### Developing pSCB-YFP as an Expression Vector (pSCBe)

**FIGURE 3:**
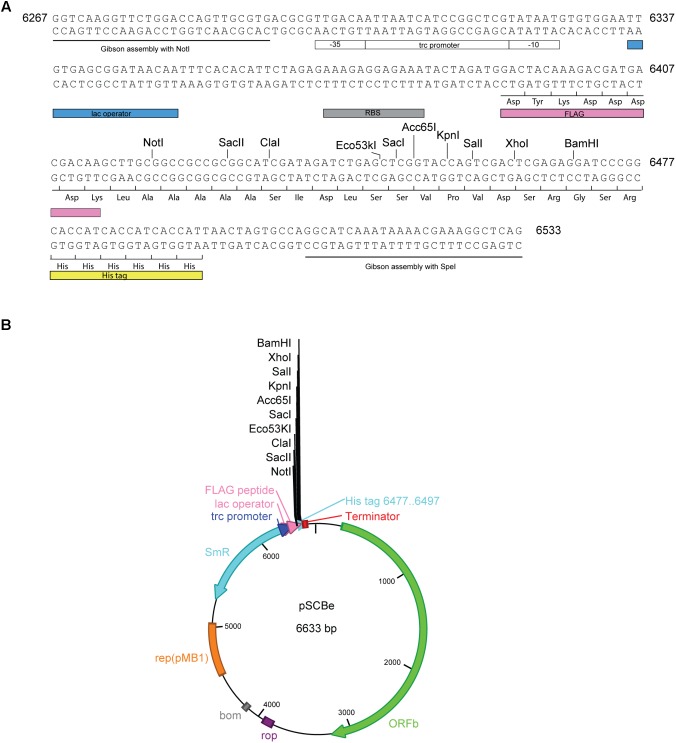
pSCBe expression vector generation. **(A)** pSCBe-MCS fragment synthesized from IDT. The fragment contains two 26 bp upstream and downstream overlap sequences for Gibson assembly; Trc1O promoter which has lac operator; RBS site; an N-terminal FLAG tag; MCS with 10 unique enzyme digestion sites when clone into NotI and SpeI digested pSCB-YFP; and a C-terminal His tag. Number 6267 to 6533 indicated fragment position on pSCBe vector. **(B)** Expression vector pSCBe graph. The detail generation procedure and property of plasmid was described in “Materials and Methods” and “Results” sections.

To develop pSCB-YFP as a vector for heterologous gene expression, the following steps were taken. A 266 bp *pSCBe-MCS* fragment was synthesized (**Figure 3A**) which contains the Trc1O promoter. In the absence of the LacI repressor, the Trc1O promoter cannot be repressed and behaves as a strong, constitutive promoter (Huang et al., 2010; Camsund et al., 2014). There is a short, hydrophilic 8-amino acid peptide (FLAG) that can be fused with recombinant protein of interest at the N-terminus. The FLAG peptide is easily accessible for cleavage by enterokinase (Ek) and for detection with antibodies. In addition, because of the small size of the FLAG peptide tag, it is not likely to obscure other epitopes, domains, alter function or transport of the fusion protein (Einhauer and Jungbauer, 2001). In addition, there is a MCS which contains 10 unique enzyme digestion sites (listed in **Figure 3B**). There is a His tag at the C-terminus of this expression vector. Both the N-terminus FLAG tag and C-terminus His tag can be used for Western blotting, immunoprecipitation, and protein purification. The pSCBe expression vector (**Figure 3B**) was verified by sequencing and deposited in Addgene’s non-profit plasmid repository under ID 110065. The plasmid sequence was also submitted to GenBank (accession number MH144609).

### pSCB-YFP Vector Maintenance Without Antibiotic Selection

**FIGURE 4:**
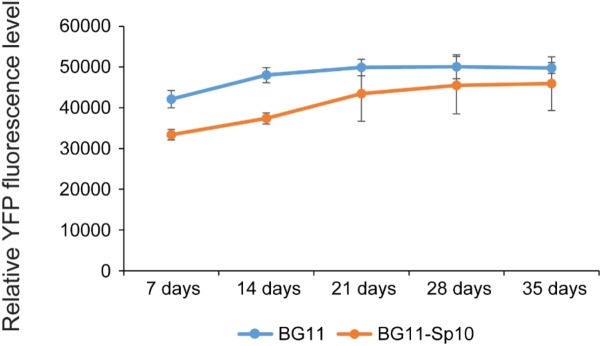
pSCB-YFP plasmid maintenance without antibiotic selection over time based on YFP fluorescence. In order to assay pSCB-YFP stability in the absence of antibiotic selection pressure, SCB-YFP exconjugants were grown in BG11 medium supplemented with 10 μg/ml Sp antibiotic for 7 days. This was used as seed culture for further inoculation into two different kinds of medium with an initial OD_730_ = 0.1. One was BG11 medium supplemented with 10 μg/ml Sp, the other was BG11 medium lacking antibiotics. These were grown under defined conditions as described in “Materials and Methods” and then assayed for YFP fluorescence. Syn6803M strain grown in BG11 medium to the same stage was used as negative control. The fluorescence assay showed that YFP fluorescence levels did not decline in any of the treatments. SCB-YFP even showed slightly higher fluorescence level in antibiotic-free medium (BG11 in blue color) than in BG11 medium supplemented with Sp10 (BG11 + Sp in orange color) during first 2 weeks. There was no significant fluorescence level difference between the two treatments during a 5-week period. Error bars represent standard derivation from three biological replicates which were taken from three independent cultures growing side by side and three technical replicates.

Based on the results described above, the pSCB-YFP plasmid can stably exist in Syn6803M and does not recombine into the host genome. Next, we asked whether the plasmid is stably maintained in the absence of antibiotic selection. This is a highly desirable trait, especially for large industrial bioengineering applications, where addition of antibiotic is prohibitively expensive. The Syn6803M strain used in this study is motile and tends to spread rapidly on plates, so it is challenging to accurately count Sp-resistant colonies after extended culturing of SCB-YFP strains in a liquid medium without Sp (Chen et al., 2016b). To overcome this, we quantified YFP fluorescence, which reflects plasmid copy number in the cell. If pSCB-YFP were not stable, cells would lose fluorescence over time. However, exconjugants showed no loss in YFP fluorescence after 35 days (>50 generations) in the absence of antibiotic selection (**Figure 4**). This indicates that the pSCB-YFP vector can persist stably even in the absence of antibiotic selection pressure. In contrast, the shuttle vector pANS designed for Syn7942, requires a toxin-antitoxin to maintain its existence (Chen et al., 2016b).

### Co-existence of pSCB-YFP and the RSF1010 Replicon Based Shuttle Vector pPMQAK1-GFP

**FIGURE 5:**
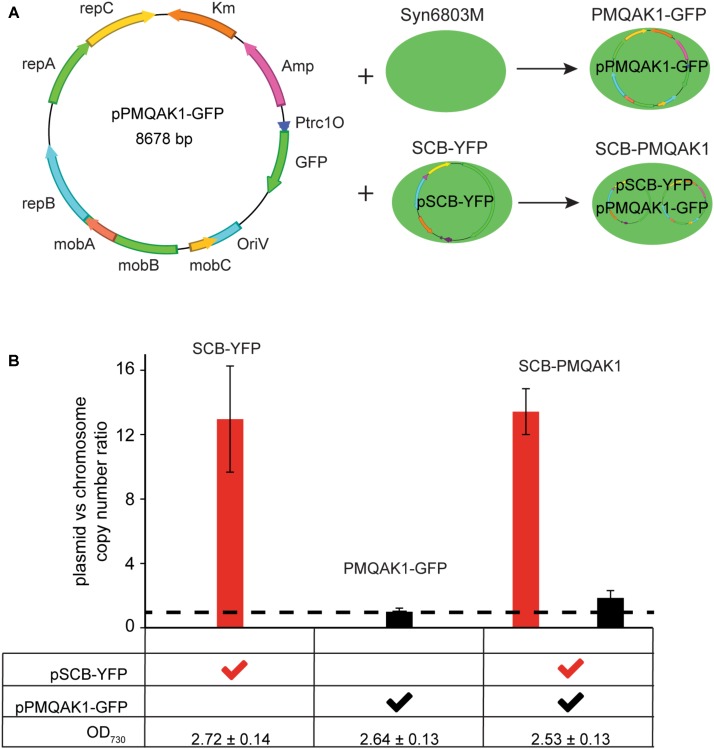
pSCB-YFP compatibility with pPMQAK1-GFP and its copy number assay. **(A)** pPMQAK1-GFP, a construct based on the RSF1010 replicon, was gifted from Huang et al. (2010). It was conjugated into Syn6803M and SCB-YFP, which generated PMQAK1-GFP and SCB-PMQAK1 exconjugants, respectively. **(B)** pSCB-YFP and pPMQAK1-GFP plasmid copy number were assayed from three different exconjugants, SCB-YFP, PMQAK1-GFP, and SCB-PMQAK1. All the cells were taken from stationary phase when the OD_730_ was ∼2.5 to 2.7. The plasmid to chromosome copy number ratio was calculated as the number on *Y*-axis. DB423 and DB424 amplicon from pSCB-YFP, and DB419 and DB420 amplicon from pPMQAK1-GFP were used as targets and single copy *petB* gene fragment from chromosome was used as a reference. pPMQAK1-GFP plasmid has a similar copy number to the chromosome (ratio ∼1; dashed line), while the pSCB-YFP plasmid copy number ratio to the chromosome is ∼10 to 13. Error bars represent standard deviations calculated from two biological and three technical replicates.

We have demonstrated that the pSCB-YFP shuttle vector can be used to express YFP in the cell. We also tested if pSCB-YFP is compatible with pPMQAK1-GFP. PMQAK1-GFP is based on the widely used broad host range replicon RSF1010 and has the GFPmut3 reporter gene driven by a Trc1O promoter (**Figure 5A**) (Huang et al., 2010). Two shuttle vectors that are compatible in Syn6803 would be useful for building complex gene circuits in cyanobacteria (Moon et al., 2012; Immethun et al., 2016). To test this, pPMQAK1-GFP was conjugated into the SCB-YFP strain, which generated SCB-PMQAK1 exconjugants containing both plasmids. These cells were resistant to Cb, Km, and Sp suggesting that both vectors were stable in the cell. So far, the SCB-PMQAK1 strain can be maintained on triple antibiotic plates for several generations without spontaneous loss of either plasmid. Meanwhile, pPMQAK1-GFP was introduced into Syn6803M by conjugation, which generated the PMQAK1-GFP exconjugants with Cb and Km resistance (**Figure 5A**).

To determine the absolute copy number of the two shuttle vectors in Syn6803M is challenging because both chromosome and plasmid copy number vary as a function of growth stage. It is known that pPMQAK1-GFP is a low copy number plasmid in *E. coli* (10–20 copies) (Sakai and Komano, 1996) and in Syn6803 (Huang et al., 2010). The copy number of pSCB-YFP in Syn6803M has not been established. To address this, we used qPCR to determine the ratio of plasmids pSCB-YFP and pPMQAK1-GFP to the chromosome. Considering that Syn6803 genome copy number is highly growth phase dependent (Griese et al., 2011) and microarray data showed pCC5.2 ORFb is one of the genes which is predicted to be expressed at the highest levels during stationary phase (Berla and Pakrasi, 2012), plasmid copy number in all exconjugants were assayed under stationary phase (5-day-old cultures, OD_730_ = 2.6 ± 0.2). We assume that at this stage, the cell number is similar in cultures with the same optical densities and that the chromosome number per cell is also similar. The results showed that pPMQAK1-GFP is a low copy number plasmid which is maintained at a similar or slightly higher copy number relative to the Syn6803 chromosome (**Figure 5B**, dash line). This is in agreement with a previous study (Huang et al., 2010). The copy number of pSCB-YFP appears to be 10 times higher than the chromosome copy number, when a single copy gene is used as the reference (*petB*) (**Figure 5B**). When 16S rRNA, which is present as two copies on the genome is used as the reference, the ratio is approximately fivefold to eightfold (Supplementary Figure 2). Considering the high level of polyploidy of the Syn6803M genome (estimated at approximately 58 copies per cell at stationary phase) (Griese et al., 2011), we concluded that the RSF1010 replicon supports higher copies in Syn6803M than in *E. coli* (10–20 copies). Based on this, we estimate that the shuttle vector pSCB-YFP is present at 350–500 copies per cell in Syn6803M, this suggests that high gene expression is likely to be supported. qPCR assay also showed that pSCB-YFP and pPMQAK1-GFP vectors can stably co-exist without interference after 10 generations in SCB-PMQAK1 strain. Actually, we have being growing SCB-PMQAK1 for at least a year in the lab and have never noticed a loss of resistance to the three antibiotics.

## Accession Numbers

Shuttle vector pSCB-YFP and pSCBe are deposited in Addgene’s non-profit plasmid repository under ID 101737 and 110065, the whole sequences of the plasmids have been submitted to GenBank (MH144608 and MH144609, respectively).

## Author Contributions

HJ conceived the study and designed the experiments. HJ and YW performed the experiments. HJ and DB analyzed the data, interpreted the results, and drafted the manuscript with contribution from AI and YW. All authors read and approved the final manuscript.

## Conflict of Interest Statement

The authors declare that the research was conducted in the absence of any commercial or financial relationships that could be construed as a potential conflict of interest.

## Footnotes

1 http://parts.igem.org/
