# Supplementary material for: Construction of a Shuttle Vector Using an Endogenous Plasmid From the Cyanobacterium Synechocystis sp. PCC6803
Source: Front Microbiol. 2018 Jul 24;9:1662. doi: 10.3389/fmicb.2018.01662 (PMC6066503; doi:10.3389/fmicb.2018.01662)
Supplement: Supplementary file 1 [file Image_1.PDF]

Gibson assembly MCS

TTCTGGACCAGTTGCGTGACGCGGCCGCCAGATCTTCCGGATGGCTCGAGTTTTTCAGCAAGAT

ATCTTACTAGAAGATCTCCTACAATATTCTCAGCTGCCATGGAAAATCGATGGTTGACAATTAA

Trc1O promoter

TCATCCGGCTCGTATAATGTGTGGAATTGTGAGCGGATAACAATTTCACACATTCTAGAGAAAG

AGGAGAAATACTAGATGGTGAGCAAGGGCGAGGAGCTGTTCACCGGGGTGGTGCCCATCCTGGT

CGAGCTGGACGGCGACGTAAACGGCCACAAGTTCAGCGTGTCCGGCGAGGGCGAGGGCGATGCC

ACCTACGGCAAGCTGACCCTGAAGTTCATCTGCACCACCGGCAAGCTGCCCGTGCCCTGGCCCA

CCCTCGTGACCACCTTCGGCTACGGCCTGCAATGCTTCGCCCGCTACCCCGACCACATGAAGCT

GCACGACTTCTTCAAGTCCGCCATGCCCGAAGGCTACGTCCAGGAGCGCACCATCTTCTTCAAG

YFP

GACGACGGCAACTACAAGACCCGCGCCGAGGTGAAGTTCGAGGGCGACACCCTGGTGAACCGCA

TCGAGCTGAAGGGCATCGACTTCAAGGAGGACGGCAACATCCTGGGGCACAAGCTGGAGTACAA

CTACAACAGCCACAACGTCTATATCATGGCCGACAAGCAGAAGAACGGCATCAAGGTGAACTTC

AAGATCCGCCACAACATCGAGGACGGCAGCGTGCAGCTCGCCGACCACTACCAGCAGAACACCC

CCATCGGCGACGGCCCCGTGCTGCTGCCCGACAACCACTACCTGAGCTACCAGTCCGCCCTGAG

CAAAGACCCCAACGAGAAGCGCGATCACATGGTCCTGCTGGAGTTCGTGACCGCCGCCGGGATC

ACTCTCGGCATGGACGAGCTGTACAAGTAATAATACTAGTGCCAGGCATCAAATAAAACGAAAG

terminator

GCTCAGTCGAAAGACTGGGCCTTTCGTTTTATCTGTTGTTTGTCGGTGAACGCTCTCTACTAGA

Gibson assembly

GTCACACTGGCTCACCTTCGGGTGGGCCTTTCTGCGTTTATATAAACAGCGTGACAGCGACCG

Supplementary Figure 1.
This is the pSCB-YFP synthesis fragment from IDT. There are two 20 bp gibson assembly overlap sequence with either *aadA* gene or ORFb 5' UTR regioin. This synthesis fragment is consisting of MCS (black line); Trc1O promoter (blue line); YFP gene (yellow line); and a terminator downstream of YFP gene.

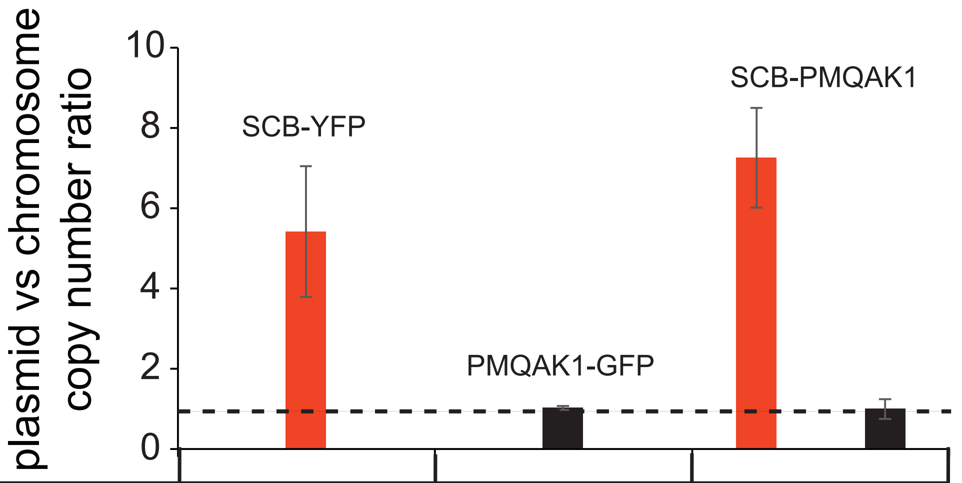

pSCB-YFP	✓		✓
pPMQAK1-GFP		✓	✓
OD_{730}	2.72 ± 0.14	2.64 ± 0.13	2.53 ± 0.13

Supplementary Figure 2.
pSCB-YFP and pPMQAK1-GFP plasmids copy number ratio to 16sRNA gene which has two copies located on chromosome. The assay was conducted on all three different exconjugants, SCB-YFP, PMQAK1-GFP, and SCB-PMQKA1. All the cells were taken from stationary phase which OD_{730} were around 2.5 to 2.7, the plasmid to chromosome copy number ratio was calculated as number on Y-axis. DB423 & DB424 amplicon from pSCB-YFP, and DB419 & DB420 amplicon from pPMQAK1-GFP were used as target and 16sRNA gene fragment from chromosome was used as reference. pPMQAK1-GFP plasmid has similar copy number as chromosome which the ratio around 1 (dash line), while pSCB-YFP plasmid copy number ratio to chromosome is around 5 to 7 times. Error bars represent standard deviations calculated from two biological and three technical replicates.
